# Multi-institutional study of ‘Sandwich treatment’ for motor area large brain metastases (LBM) with diameter over 3 cm

**DOI:** 10.2478/raon-2024-0002

**Published:** 2024-01-06

**Authors:** Zheng Wang, Haining Chen, Qun Chen, Yucun Zhu, Min Li, Jia Zhou

**Affiliations:** Cancer Center, Gamma Knife Treatment Center, Zhejiang Provincial People’s Hospital, Affiliated People’s Hospital of Hangzhou Medical College, Hangzhou, China; Gamma Knife Treatment Center, Anhui Provincial Hospital, The First Affiliated Hospital of University of Science and Technology of China, Hefei, China; Gamma Knife Treatment Center. Jiangsu Province People’s Hospital, the First Affiliated Hospital of Nanjing Medical University Nanjing, China; Gamma Knife Treatment Center, Ming ji Hospital, Affiliated to Nanjing Medical University, Nanjing, China

**Keywords:** Sandwich treatment, bevaczumab, two-staged SRS, motor area, large brain metastases

## Abstract

**Background:**

The objective of the present study was to explore the effectiveness and safety of ‘Sandwich treatment’ strategy for large brain metastases (LBM) with diameter over 3 cm (minimum volume >= 15 cm^3^) located in motor area.

**Patients and methods:**

Patients from four gamma knife center that received ‘Sandwich treatment’ were retrospectively studied from January 2016 to March 2023. The strategy was one-week treatment course including 2 stages of stereotactic radiosurgery (SRS) and using bevacizumab once during SRS gap. The tumor volume and peri-tumor edema changes were analyzed before and after ‘Sandwich treatment’. Manual muscle testing (MMT) score and Barthel Index (BI) score were used to evaluate the changes of patients’ movement and physical strength rehabilitation. The patients’ overall survival (OS) and tumor local control (TLC) rate was calculated. Cox regression model was used to analyze the risk factors that related to TLC.

**Results:**

61 patients with 72 lesions received the ‘Sandwich treatment’. The median prescription dose was 13.0 Gy and 12.5 Gy at the first- and second-stage SRS. The mean tumor volume at the time of ‘Sandwich treatment’ and 3 months later was 20.1 cm^3^ and 12.3, respectively (P < 0.01). The mean peri-tumor edema volume at the first- and second-stage SRS was 12.6 cm^3^ and 5.2 cm^3^, respectively (P < 0.01). Patients’ median MMT score improved from 6 at the beginning to 8 at the end of ‘Sandwich treatment’ (P < 0.01), BI score was also greatly improved from 45 at the time of ‘Sandwich treatment’ to 95 after 3 months (P < 0.01). Patients’ median OS was 14.0 months, and the 3, 6, 12 months OS rate was 92.0%, 86.0% and 66.0%, respectively. The TLC rate at 3, 6, 12 months was 98.4%, 93.4%, and 85.3%, respectively. Patients with lung cancer had lower risk of tumor relapse. The cumulative incidence of patient’s hemorrhage and radiation necrosis was 4.92% (3/61) and 13.11% (8/61) after ‘Sandwich treatment’.

**Conclusions:**

‘Sandwich treatment’ strategy is safe and effective for LBM located in motor area. The strategy could rapidly improve the patients’ movement and enhance their physical strength rehabilitation.

## Introduction

Brain metastases (BM) is the most common intracranial malignant tumor in adults, and is also the main cause of mortality of cancer patients.^1^ The current guidelines suggest that patient with limited number of BM with good performance can be treated with stereotactic radiosurgery (SRS) alone.^2,3,4,5^ For patients with BM number less than 10, or the total volume smaller than 4 cm^3^, fractioned radiosurgery or two-staged stereotactic radiosurgery (2-SSRS) could control the tumor growth with low neurotoxicity and not delay the consequent systemic treatment.^6,7,8,9,10,11^

However, for large BM (LBM) with diameter over 3 cm (minimum volume >= 15 cm^3^) and located in motor area, even 2-SSRS is still challenging.^12^ The risk mainly comes from the compression of LBM and consequent peri-tumor edema to the brain, results in devastating intracranial hypertension. Further, the SRS-induced edema would add to the risk of intracranial hypertension, limb hemiplegia and refractory epilepsy.^13,14^ Meanwhile, SRS would cause the brain radiation necrosis (RN).^15,16,17^ These potential risks make it difficult for patients with LBM to receive 2-SSRS in outpatient department. Patient after SRS needs long-term inpatient steroids therapy to control peri-tumor and SRS-induced edema to improve their symptoms, and lower the potential risk of RN. Bevacizumab, an anti-VEGF monoclonal molecular drug, has been utilized by practitioners in anti-tumor therapy for cancer patients.^18,19^ Also, its anti-angiogenesis effect could be used for SRS-edema and RN control.^20,21^ To help patient with motor area LBM and shorten their treatment course, the practitioners from four gamma knife center developed the ‘Sandwich treatment’ strategy. The strategy was one-week treatment course that includes 2-SSRS and using bevacizumab once during SRS gap. In the present study, the authors retrospectively reviewed the patients that received ‘Sandwich treatment’. The purpose of this study was to evaluate the efficacy of and safety of this strategy.

## Patients and methods

### Patients

From January 2016 to March 2023, patients with LBM that received the ‘Sandwich treatment’ were retrospectively studied. The inclusion criteria are as follows: patients had (1) at least one newly diagnosed BM in the motor area; (2) tumor diameter larger than 3 cm and no previous whole brain radiation therapy; (3) received steroid therapy for tumor or peri-tumor edema controlling; (4) not ongoing systemic therapy. Because the retrospective observation study was focused mainly on the effectiveness and safety of ‘Sandwich treatment’ for LBM in motor area, patients fitted the inclusion criteria were all included despite they received treatment for previous primary tumor or not. The present study was approved by the Institutional Ethics Committee of Zhejiang Provincial People’s Hospital (ZHRYRS 2022 No. 005).

### ‘Sandwich treatment’ strategy

The ‘Sandwich treatment’ was a one-week treatment course. Two-SSRS were delivered to patients with mean dose of 13 Gy and 12.5 Gy at first and second SRS respectively. At each stage of SRS, the 45% – 60% isodose line covered the whole lesion. The bevacizumab was used once 3 days later after the first-stage SRS, for the purpose of tumor growth and peri-tumor edema control. The dose of bevacizumab was 5 mg/kg according to previous studies had suggested of 5–10 mg/kg.^22,23^ Target volumes were obtained from Gadolinium enhanced T1-weighted magnetic resonance images (MRI). The edema volume was accessed from MRI T2-FLAIR.

### Evaluation of efficacy and adverse events

Tumor local control (TLC) failure was defined as 20% increase in product of perpendicular diameter on T1-enhanced MR after ‘Sandwich treatment’ according to the revised RANO guidelines.^24^ Radiation necrosis (RN) was determined MRI perfusion and PET results and clinical symptoms.^25,26^ The manual muscle testing (MMT) score was used for the evaluation of patients’ muscle strength change.^27^ The scale proposed by the Medical Research Council (MRC) uses the numeral grades 0–5, 0: No contraction; 1) Flicker or trace contraction; 2) Active movement, with gravity eliminated; 3) Active movement against gravity; 4) Active movement against gravity and resistance; 5) Normal power. The total score of upper limb and lower limb was summed up as the baseline standard for the evaluation of patients’ muscle strength. The Barthel Index (BI) is used to assess patients’ early rehabilitation after radiosurgery.^28^ Overall survival (OS) was defined as the time interval from patients finished the ‘Sandwich treatment’ to their death.

### Statistical analysis

Follow-up time were defined as the time from completion of ‘Sandwich strategy’ to the time of most recent follow-up. End-point events were illustrated using Kaplan-Meier method. Categorical data were presented as percentages and compared by Mann-Wittney U test. Continuous data using t-test. Cox regression model was used to analyze the risk factors that related to TLC. All statistical analyses were performed using SPSS version 19.0 (IBM Corp., Armonk, New York) or GraphPad Prism 8.0 (La Jolla, California, United States). Values with P < 0.05 were considered statistically significant.

## Results

### Patient characteristics

A total of 61 patients with 72 LBM located in the motor area received the ‘Sandwich treatment’ from January 2016 to March 2023. 36 patients were female and 35 were male. Patients’ median age was 62 years (range: 34–81 years). The median Karnofsky performance status (KPS) score before treatment was 60 (range: 50–80). The median dose at the first and second SSRS was 13 Gy (range: 11–15 Gy) and 12.5 Gy (range: 11–14 Gy), respectively. The median follow-up time of 61 patients was 18.3 months (range: 6.3–47.9 months). None of the treated patients had accidental intracranial hemorrhage after bevacizumab treatment. Detailed general patient characteristics are presented in Table 1.

**TABLE 1.** Patient characteristics

**Characteristic**	**Value**	**Range**
Age(median, years)	62	34–81
Sex		
Female	36	
Male	25	
Primary tumor		
Lung	39	
Breast	13	
Gastric-intestinal tract	9	
KPS (median)	60	50 –80
Dose at first-stage SRS (median, Gy)	13.0	11–15
Dose at second-stage SRS (median, Gy)	12.5	11–14
Total tumor volume (mean, cm^3^)	20.1	17.2–29.7
Peri-tumor edema volume (mean, cm^3^)	12.6	4.9–19.6
Follow-up time (median, months)	18.3	6.3–47.9

KPS = Karnofsky performance status

### The volume changes of tumor and peri-tumor edema

**FIGURE 1.**
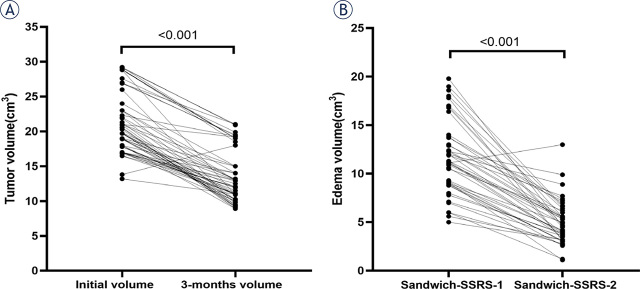
The mean tumor and peri-tumor edema volume changes. **(A)** The mean tumor volume decreased dramatically from 20.1 cm^3^ (range: 17.2–29.7 cm^3^) at the time of ‘Sandwich treatment’ to 12.3 cm^3^ (range: 7.7–22.4 cm^3^) 3 months later (P < 0.001); **(B)** The mean peri-tumor edema volume at first-stage SRS and second-stage SRS of ‘Sandwich treatment’ was 12.6 cm^3^ (range: 4.9–19.6 cm^3^) and 5.2 cm^3^ (range: 1.2–13.2 cm^3^) (P < 0.001). 2-SSRS = two-staged stereotactic radiosurgery

**FIGURE 2.**
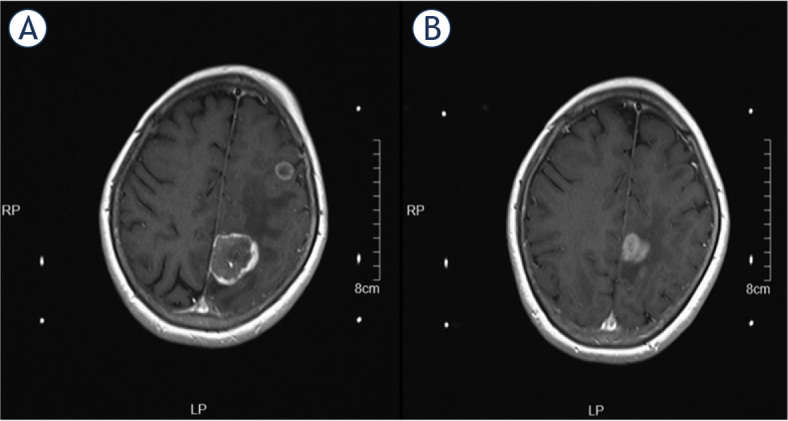
A typical case with LBM located in motor area that received ‘Sandwich treatment’. **(A)** The lesion had severe peri-tumor edema and invading towards the frontal lobe; **(B)** 3 months after ‘Sandwich treatment’, peri-tumor edema and tumor volume significantly reduced.

There was no statistical tumor volume change at first-stage SRS and second-stage SRS during the ‘Sandwich treatment’. However, the mean tumor volume decreased dramatically from 20.1 cm^3^ (range: 17.2–29.7 cm^3^) at the time of ‘Sandwich treatment’ to 12.3 cm^3^ (range: 7.7–22.4 cm^3^) 3 months later (P < 0.001, Figure 1 A). The mean peri-tumor edema volume at the 1-SSRS and the 2-SSRS of ‘Sandwich treatment’ was 12.6 cm^3^ (range: 4.9–19.6 cm^3^) and 5.2 cm^3^ (range: 1.2–13.2 cm^3^), with significant statistical difference (P < 0.001, Figure 1 B). Figure 2 shows a patient that received ‘Sandwich treatment’ for LBM located in motor area, the peri-tumor edema and tumor volume significantly reduced 3 months later.

### Patients’ MMT score and BI score changes

**FIGURE 3.**
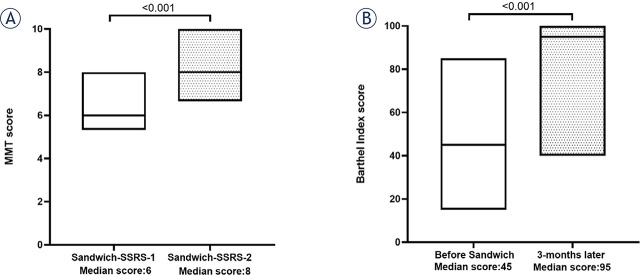
Patients’ median MMT and BI score. **(A)** MMT score at the beginning and the end of ‘Sandwich treatment’ (P < 0.001); **(B)** BI score before ‘Sandwich treatment’ and at 3 months later (P < 0.001). BI score = Barthel Index score; MMT score = manual muscle testing score; 2-SSRS = two-staged stereotactic radiosurgery

Patients’ median MMT score improved from 6 (range: 5–8) at the beginning to 8 (range: 7–10) at the end of ‘Sandwich treatment’ (P < 0.001, Figure 3 A). Patients’ median BI score was also greatly improved from 45 (range: 15–85) at the time of ‘Sandwich treatment’ to 95 (range: 40–100) after 3 months (P < 0.001, Figure 3 B).

### Patients’ OS, TLC and prognostic factors for TLC

**FIGURE 4.**
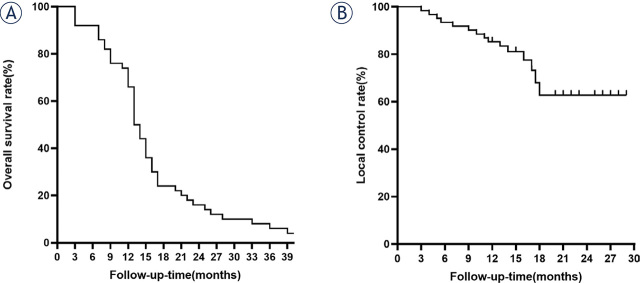
Kaplan - Meier curve of the patient’s **(A)** Overall survival (OS) and **(B)** tumor local control (TLC) rate.

As Kaplan - Meyer curve showed in Figure 4A, the patient’s median survival time was 14.0 months, and the overall survival rates at 3, 6, 12 months was 92.0%, 86.0% and 66.0%, respectively. The TLC rate at 3, 6, 12 months was 98.4%, 93.4%, and 85.3%, respectively (Figure 4B). Primary tumor types (Lung/Breast/GI tract cancer) were prognostic factors for TLC in Univariate analysis. Multivariate analysis revealed that patients with lung cancer had lower risk of tumor relapse [Lung/Breast: HR = 0.539, 95% CI:(0.339–0.812); Lung/GI tract: HR = 0.784, 95%CI:(0.498–0.987)] (Table 2).

**TABLE 2.** Risk factors of TLC for LBM in motor area after ‘Sandwich treatment’

	**Univariate analysis**			**Multivariate analysis**		

	**HR ratio**	**95%CI**	**P-value**	**HR ratio**	**95%CI**	**P-value**
**Age**	0.978	(0.598–1.686)	0.697			
>=62						
<62						
**Sex**	1.678	(0.913–2.174)	0.781			
Male						
Female						
**Primary tumor**						
Lung/Breast	0.459	(0.359–0.898)	< 0.01	0.539	(0.339–0.812)	0.007
Lung/GL tract	0.913	(0.478–1.316)	< 0.01	0.784	(0.498–0.987)	0.006
Breast/Gl tract	1.987	(0.878–1.974)	0.411			
**KPS score**	2.113	(1.159–6.733)	0.139			
>= 60						
<60						
**Total tumor volume**	0.719	(0.419–1.598)	0.211			
>=20.1cm^3^						
<20.1cm^3^						
**Peri-tumor edema volume**	1.589	(0.973–2.894)	0.566			
>=12.6cm^3^						
<12.6cm^3^						
**Dose at first-stage SRS**	1.325	(0.53–1.734)	0.989			
>= 13.0 Gy						
< 13.0 Gy						
**Dose at second-stage SRS**	0.845	(0.356–1.250)	0.593			
>= 12.5 Gy						
< 12.5 Gy						

KPS = Karnofsky performance status; LBM = large brain metastases; 2-SSRS = two-staged stereotactic radiosurgery; TLC = tumor local control

### Side effects of ‘Sandwich treatment’

The side effects of ‘Sandwich treatment’ mainly consist of hemorrhage hazard by bevacizumab and radiation necrosis (RN) by SRS. However, no patients had accidental intracranial hemorrhage after ‘Sandwich treatment’, and the cumulative incidence of hemorrhage was 4.92% (3/61), mainly oral and nasal bleeding. The cumulative radiation necrosis was 13.11% (8/61) in all patients, only 3.3% (2/61) presented with symptoms.

### Study limitations

This study has several limitations. Firstly, the number of patients is not large and would interfere the results of statistical analysis. Secondly, patients received ‘Sandwich treatment’ may receive different consequent systemic therapy, so the influence weight of these different therapies on the local control of BM cannot be precisely accessed. However, it cannot be avoided in other similar researches and commonly exists in the treatment cause of patients. Finally, there are still disputes about the personalized dose of bevacizumab for edema control after SRS. A larger cohort and prospective studies are needed to provide more thrilling results.

## Discussion

In recent years, SRS has been recognized as effective alternative treatment for BM.^2,3,4^ For BM with diameter at 2–3 cm, fractionated or staged SRS is preferable.^9,11,29^ One of the representative study on 2-SSRS method for BM is reported by Angelov *et al.* in 2018.^10^ In their case series, the volume of 63 BMs in 54 patients reduced significantly, and the TLC rate in 3, 6 months after treatment reaches 95% and 88%, respectively; the incidence of overall radiation side effects was 11%. Another representative study was conducted by Serizawa T *et al.*^11^ They compared the treatment results between 3- and 2-stage Gamma Knife radiosurgery for large BM and found no differences between in terms of patients’ overall survival, tumor progression, neurological death, and radiation-related adverse events. Dohm Amoren *et al.* reported 2-SRSS for BM that are difficult to be removed by surgery^8^, the cumulative incidence of local treatment failure at 6 and 12 months was 3.2% and 13.3% respectively. The study of Damron *et al.* in 2022 also supports the effectiveness and safety of 2-SSRS for BM patients.^30^ According to the results of these studies, 2-SSRS for the BM treatment is satisfactory and local control failure rate is low. Table 3 listed 8 studies that adopted 2-SSRS strategy for BM.

**TABLE 3.** Comparison between ‘Sandwich treatment’ and other reports of 2-SSRS strategy for BM

Author	Year	Case number	Median diameter/volume of BM	Total dose	2-SSRS interval	6 Months OS/median OS	6 Months TLC/median TLC	Radiation necrosis
Yomo *et al.*^9^	2012	27	17.8 cm^3^	27 Gy	3−4 weeks	8.8 months	89.8%	11.1%
Yomo and Hayashi^29^	2014	58	16.4 cm^3^	28 Gy	3−4 weeks	63%	85.0%	8.6%
Angelov *et al.*^10^	2018	54	Diameter>=2cm	30 Gy	34 days	65%	88.0%	11.0%
Dohm *et al.*^8^	2018	33	LBM	29 Gy	30 days	65%	96.8%	6.06%
Hori *et al.*^31^	2020	181	4 cm^3^	N/A	N/A	14.6 months	91.0%	N/A
Ito *et al.*^32^	2020	178	10 cm^3^	26 Gy	7−38 days	6.6 months	93.2%	6.20%
Damron *et al.*^30^	2022	24	8.1 cm^3^	30 Gy	32 days	9.1months	80%	N/A
Cho *et al.*^33^	2022	142	Median 7.4 cm^3^	27−28 Gy	32 days	14 months	88−90%	17%
Present study	2023	51	20.1 cm^3^	25.5 Gy	7 days	91.8%, 14 months	93.4%	13.11%

N/A = not available

BM = brain metastases; 2-SSRS = two-staged stereotactic radiosurgery

The listed studies mainly focused on the optimal prescription doses of the 2-SSRS. The researchers suggested dose reduction strategy was suitable for lessening of SRS-induced edema and RN. However, the 2-SSRS strategy might not be enough for the LBM located in motor area. Meanwhile, these conventional 2-SSRS strategy have treatment course longer than 1 month. Patients may have to receive even longer time of inpatient dehydration and steroid treatment during the 2-SSRS when the diameter of BM was over 3 cm and located in the motor area. The long-term usage of mannitol and steroid hormones would bring a series of side effects to the patient, delay their consequent systemic treatment, increase their treatment cost. On the other hand, patient’s intracranial hypertension, neuro-dysfunction symptoms and the risk of suffering from refractory epilepsy would make them hardly to receive SRS at outpatient department.

The side effect of 2-SSRS should not be neglected as well. The SRS-induced edema would aggravate the edema caused by LBM compression in the motor area, patients would experience deterioration of limb movement and their life quality. Cho *et al.* pointed out even 2-SSRS strategy for BM could cause RN as high as 17% and affects the life quality of patients.^33^ Considering these tough issues, we introduce bevacizumab. Juan *et al.* had applied bevacizumab for the treatment of SRS related edema, and found the edema volume reduction at 49.0%–66.0% on MRI-T2FLAIR.^34^ As bevacizumab could reduce the angiogenesis around the lesion, it could also lower the brain RN risk.^35^ Its anti-angiogenesis effect could be used to control tumor growth had synergies with SRS. Therefore, the ‘Sandwich treatment’ strategy would have obvious advantage in the treatment of LBM in motor area, especially for those with primary lung adenocarcinoma.^36^

The results of this retrospective study confirmed the effectiveness and safety of ‘Sandwich treatment’. The results indicated that using bevacizumab during SRS gap could reduce the median volume of peri-tumor edema by 38.8% (from 12.6 cm^3^ to 5.2 cm^3^). Meanwhile, patient’s muscle strength score that reflecting the patients’ physical activities also significantly improved. The strategy would shorten the whole treatment course while preserve patients’ neuro-function or improve their life quality. Patients with primary lung adenocarcinoma had significant lower risk of tumor relapse. Compared to previous 2-SSRS reports as showed in Table 3, the present study had the largest median tumor volume, shortest treatment course, while the TLC were similar compared to other studies. The 6-month OS rate and median survival in our present study exceeded 6 of the 8 studies.

The incidence of RN in this study was higher than other eight 2-SSRS cohorts listed in Table 3. The main reason may be that in these cohort the patient’s median OS was short and they failed to report the potential RN symptoms. On contrary, the LBM in the present study were all located in the motor area, and patients’ SRS-induced symptoms could be much more obvious.

## Conclusions

As far as we know, this is the first report of ‘Sandwich treatment’ strategy for the LBM with diameter over 3 cm at motor area. The statistical results verified the effectiveness and safety of this strategy. This strategy could significantly improve patients’ life quality and greatly shortened treatment interval. However, a larger cohort is still needed for prospective study.
